# Clustering and correlates of screen-time and eating behaviours among young children

**DOI:** 10.1186/s12889-018-5698-9

**Published:** 2018-06-18

**Authors:** Natalie Pearson, Stuart J. H. Biddle, Paula Griffiths, Julie P. Johnston, Emma Haycraft

**Affiliations:** 1 School of Sport, Exercise & Health Sciences, National Centre for Sport & Exercise Medicine, Loughborough University, Loughborough, Leicestershire LE11 3TU UK; 2 Institute for Resilient Regions, University of Southern Queensland, Springfield, Australia; 3 Department of Sport Science, School of Science and Technology, Nottingham Trent University, Nottingham, UK

**Keywords:** Clustering, Screen-time, Fruit, Vegetables, Energy-dense snacks, Children, Correlates

## Abstract

**Background:**

Screen-time and unhealthy dietary behaviours are highly pervasive in young children and evidence suggests that these behaviours often co-occur and are associated. Identifying clusters of unhealthy behaviours, and their influences early in childhood, can assist in the development of targeted preventive interventions. The purpose of this study was to examine the sociodemographic, behavioural, and home physical environmental correlates of co-occurring screen-time and unhealthy eating behaviours and to assess the clustering of screen-time and unhealthy dietary behaviours in young children.

**Methods:**

Parents of 126 children, from the UK, aged 5–6 years (49% boys) completed a questionnaire which assessed their child’s screen-time (ST), fruit and vegetable (FV), and energy-dense (ED) snack consumption. Categories of health behaviours were created based on frequencies of children meeting recommendations for FV and ST and median splits of frequencies for ED snacks. Parents reported on their own behaviours (ST, FV, and ED snack consumption), how often they ate meals and watched TV with their child, and on the availability and accessibility of foods within the home. An observed over expected ratio (O/E) was used to assess behavioural clustering. Multivariable multinomial logistic regression was used to examine correlates of behaviour patterns.

**Results:**

Approximately 25% of children had two or three health risk behaviours. Correlates consistently associated with clusters included parental income, eating meals at the TV, parental ST and ED snack food consumption, and home availability of ED snack foods. Observed over expected ratios were close to 1 and ranged from 0.78 to 1.43. The three-risk behaviour combination of insufficient FV consumption, high ED snack consumption, and excessive ST occurred more frequently than expected (1.23 (95% CI 0.89, 1.58)).

**Conclusions:**

ST and unhealthy dietary behaviours cluster in children as young as 5 years of age and parents’ own behaviours appear to be important influencing factors. Further research into the development of behavioural clustering in young children to identify and further understand the mechanisms underlying the synergy among health behaviours is needed. Feasibility interventions promoting reductions in both screen-time and unhealthy dietary behaviours reciprocally, while simultaneously focusing on changing parental behaviours, are warranted.

**Electronic supplementary material:**

The online version of this article (10.1186/s12889-018-5698-9) contains supplementary material, which is available to authorized users.

## Background

Unhealthy eating behaviours and too much time spent being sedentary using screen-media are highly prevalent in today’s society [1, 2]. For example, in the United Kingdom (UK), children as young as 5 years of age are using screens (television (TV), computers and tablets) for more than 27 h a week [1]. Furthermore, less than 8% of primary school-aged children meet the UK recommendations for fruit and vegetable (FV) intake [2], yet more than 13% of 4–10 year olds’ food energy comes from extra sugars [2]. A recent report from the Children’s Food Trust suggested that 4–7 year-olds were the most likely age group to eat cakes and biscuits, sweets and chocolate at least once per day (often more) [3]. Health-related behaviours, such as screen-time (ST) and unhealthy dietary behaviours, are established in childhood and tend to track throughout adolescence and into adulthood [4, 5] and so it is important to try to promote the establishment of healthy behaviours from the early years.

A wealth of evidence exists regarding the adverse health consequences associated with eating unhealthy diets (too few FV; too many energy-dense foods (ED); for example [6–8]) and spending too much time on screen-based sedentary activities (e.g. sitting to watch TV/DVDs, using computers, etc. [9, 10]). This evidence has tended to focus on these health risk behaviours independently but emerging evidence suggests that they might actually cluster and co-exist in young people [11, 12]. ST and unhealthy dietary behaviours have been found to cluster in 9–10-year-old [13] and 11–12-year-old [12] British children. Specifically, 11–12-year-old adolescents who consumed low levels of FV also consumed higher levels of ED snack foods and spent more time using screens [12]. Thus, it is plausible that health-promotion interventions, such as to prevent obesity and later ill-health, might be more effective by targeting a combination of behaviours, rather than just one.

The development of effective interventions requires an understanding of the most important correlates and determinants of the targeted behaviours. In young people, the home environment is key for health behaviours, with parents’ own behaviours and parenting strategies and practices having a significant influence on children’s health behaviours [14–16]. For example, family TV time, rules for screen-time, and access to screens at home are significant correlates of children’s ST [14]. Furthermore, parental modelling of eating FV and ED snack foods are significant correlates of child consumption of these food items, as are factors such as home availability and accessibility of food items, restriction of foods, and eating in front of the television [15, 16]. While research has typically focused on correlates of individual health behaviours there has been a recent trend towards the study of correlates of clustered behaviours [11–13, 17], however, much of this research has focussed on sociodemographic predictors [13, 17]. A recent study among 11–12 year old children examined a wider range of correlates and found numerous factors consistently associated with the co-occurence of high ST, high ED snack consumption and low FV consumption [12]. These included eating while watching TV, eating at the TV with parents, and the availability and accessibility of ED snack foods at home [12].

Given evidence that the odds of having multiple risk behaviours increase over the course of development [18], and that healthy habits are established early in life, there is value in exploring younger children, before health behaviours become too engrained. However, a recent review aiming to identify clustered patterns of diet, physical activity and sedentary behaviour among children or adolescents found only one study among children < 9 years of age [11]. In this one study, Cameron et al. [19] found that clusters of healthy (e.g. physically active and high FV) and unhealthy behaviours (low physical activity, high ST and low FV consumption, and consumption of ED food/drink) were concordant in mothers and their children (mean age 9.4 years), particularly those defined by sedentary behaviors and consumption of ED food and drink. Further evidence is required examining correlates of co-occurring behaviours and how health behaviours cluster in younger children.

Based on the identified gaps in the extant research literature, the purpose of this study was to examine the sociodemographic, behavioural, and home physical environmental correlates of co-occurring screen-time and unhealthy eating behaviours and to assess the clustering of screen-time and unhealthy dietary behaviours in young children. Pursuant to the distinctions used in epidemiological research between co-occurrence and clustering [20, 21], throughout this manuscript co-occurrence describes the concurrent, but independent, engagement in two or more health related behaviours (also referred to as patterns of health behaviours or prevalence of behaviour combinations). Clustering refers to an underlying association between co-occurring behaviours.

## Methods

### Study procedure

Following ethical approval from Loughborough University’s Ethical Advisory Committee, cross-sectional data were collected between May 2013 and June 2014. Data were obtained from parents of young children in their first year (Year 1) of primary school (aged 5–6 years) recruited from six primary schools in the East Midlands region of the UK. All eligible families were invited to take part via an information leaflet that contained details of the study. This letter was sent home for a parent or guardian (*n* = 422) and active consent was sought from parents for each child’s participation. No information was available regarding the characteristics of non-responders. In total, 233 parents provided written consent (55% response rate). Parents who consented to take part in the study received a package containing a questionnaire for completion at home.

### Measures

#### Parent questionnaire

All data were provided by the child’s main caregiver who completed a questionnaire about themselves and their child, at home. Parents’ age, gender, ethnicity, marital status, highest level of education (of the respondent), average household income and their social status (using a subjective socioeconomic status scale [22]) were reported by parents along with parents’ relationship to the participant child, and child gender and age. Demographic characteristics of parent respondents are detailed in Additional file 1: Table S1.

##### Child screen-time

Parents reported the time (in hours and minutes) that their child spent watching TV and watching videos/DVDs on a usual school day and on a usual weekend day using an adaptation of the Adolescent Sedentary Activity Questionnaire (ASAQ) [23, 24]. We adapted the ASAQ by providing parents with a category of ‘school day’ and ‘weekend day’ rather than each day separately. Time spent watching TV and watching videos/DVDs was converted into minutes per school day and weekend day respectively. Weighted mean duration ((5*schoolday+ 2*weekend day)/7) was derived and summed to provide a measure of ST. Children were classified into high or low ST groups based on the established guidelines of ≤ 2 h screen viewing [25].

##### Child eating behaviours

Parents reported how frequently their child consumed ten FV items (apples, bananas, oranges, grapes and other fruit, carrots, peas, broccoli, salad and other vegetables), and eight ED snack food items (potato crisps, snack crackers, sweets (candy), chocolate, chocolate biscuits, regular biscuits, muffins/cakes, cereal bars) during a usual week, ranging from ‘never’ to ‘more than three a day’, using a Food Frequency Questionnaire (FFQ) [26]. Using an established method [27], the frequency of consumption of the 18 food items during a typical week were converted to a daily equivalent [28, 29]. Daily equivalents were: never (0·00 per d); one-two days a week (0·2 per d); 3–4 days a week (0·5 per d); five-six days a week (0·7 per d); once a day (1.0 per d); twice a day (2.0 per d); three or more a day (3.0 per d). The daily equivalence of the food items in each group were summed to create daily intakes of FV, and ED snacks respectively. The national UK guidelines of five FV per day was used to categorise children as meeting the guidelines [30] (high or low FV). As there are no current guideline for the consumption of ED snack foods, the frequency of consumption of ED snacks were split at the median (1.6 per day) to create a high and low ED category.

##### Behavioural factors

Child frequency of consumption of meals (breakfast and dinner) and snacks (ED snacks, and FV) while watching TV were reported by parents using a previously used questionnaire by Matheson et al. [31]. Parents reported the frequency during a typical week on a four-point Likert scale ranging from (1) ‘Never’ to (4) ‘every day’. The frequency of consumption of the meals and snacks while watching TV was coded as ‘2 or less days a week’ and ‘3 or more days a week’.

Parents time spent sitting at the T*V*/*w*atching DVDs on a usual week day and a usual weekend day was assessed using the domain-specific sitting questionnaire [32]. Categories of high and low TV/DVD viewing were calculated as described above for child TV/DVD viewing.

Parents self-reported their own frequency of consumption of ED snacks by completing the same FFQ as described above. The FFQ was coded and categorised identically to that of the child (see above) but using a median frequency of consumption of 1.4 per day to create a high and low ED category.

##### Physical environmental factors

Parents reported on the availability of ED snacks (four questions) and the availability of FV (two questions) in the home during the past week. Parents responded on a four-point Likert scale ranging from (1) ‘Never/rarely’ to (4) ‘Always’. High and low availability of ED snacks and fruit and vegetables were determined by summing questions for home availability of ED snacks (Cronbach’s α = 0.77) and home availability of FV (Cronbach’s α = 0.82) respectively and using a median split.

### Statistical analysis

All data management and analyses were conducted using Stata V12 (Stata, College Station, TX). Co-occurring health behaviour categories were created and coded based on met/unmet guidelines: 0: one or no risk behaviours; 1: Low FV / High ED; 2: High ST / Low FV; 3: High ST / High ED; 4: 3 risk behaviours. Demographic, behavioural, and home physical environmental variables associated with the likelihood of being in each of the behavioural clusters were examined using Multinomial logistic regression analyses (The ‘one or no risk behaviours’ category was used as a referent). Variables at each level that were significantly associated with combinations of risk behaviours in the univariate multinomial logistic regression analyses were simultaneously entered into multivariable multinomial logistic regression models.

The ratio of observed number to expected number (O/E) was applied to estimate the degree of clustering of health risk behaviours and has been described elsewhere [12, 13]. The observed number (O) is the number of participants that did or did not meet guideline levels for each health behaviour divided by the total number of participants. The expected number (E) for each health behaviour was determined by the proportion of participants not meeting a specific guideline multiplied by the proportion of participants that met the guidelines for all remaining behaviours (e.g. the proportion of children that had high ST multiplied by the proportion that had low ED snack consumption, and the proportion that had high levels of FV intake). The expected prevalence for multiple health behaviours was calculated by multiplying the proportion of participants that did not meet guideline levels for a specific set of behaviours with the proportion that met guideline levels for the remaining behaviours. The difference between the observed and the expected prevalence (O/E) was calculated to examine whether the behaviours of interest co-occurred at a higher or lower rate than would be expected if there was no association between the health behaviours. Bootstrap techniques were used to calculate 95% confidence intervals. Clustering is indicated when observed over expected ratios are greater than one.

## Results

### Sample characteristics

Questionnaires were returned by 149 parents (64% of those who agreed to take part). Due to incomplete ST and/or FFQ data, 23 sets of data were excluded from the analyses. The analyses presented here are based on data for 126 children: 62 boys (mean age 5.66 (SD: 0.79) years) and 64 girls (mean age 5.50 (SD: 0.67) years). Responding parents were mostly mothers (81.9%) with a mean age of 38.61 (SD: 4.97) years. The majority of parents were married (80.5%) and of White/White British ethnicity (80.5%). Over two-thirds (68.9%) of parents had completed at least degree level education, and over three-quarters had an average household income of more than GBP39k income a year (Additional file 1: Table S1).

The prevalence of not meeting individual health behaviour guidelines as well as the prevalence of combinations of behaviours are described in Table 1. More than 19% of children had all three risk behaviours and 43% had none or one risk behaviour. More than 78% of children consumed less than the recommended five portions of FV per day, and 40% exceeded 2 h a day of ST.

**Table 1:** Descriptive characteristics of the child participants (*n* = 126)

	All
N (%)	126
Child age, years (mean (SD))	5.58 (0.73)
TV/DVD viewing	
> 120 min/day, %	38.8
Fruit and vegetable intake	
< 5 a day (frequency of consumption/day), %	78.6
Energy-dense snack intake	
> 1.6 a day (frequency of consumption/day), %	48.9
Risk behaviour groups (%)	
None or one risk behaviour	43
Low FV / high ED	20
High ST / low FV	12.6
High ST / high ED	5.2
3 risk behaviours (Low FV / high ST / high ED)	19.3

#### Associations with combinations of child risk behaviours

Additional file 1: Table S1 displays the description and distribution of the demographic, behavioural, and home physical environmental variables of interest.

#### Demographic factors

Of the demographic factors, only parental income was associated with behavioural risk factor combinations in the univariate analyses (Table 2). Parents of lower income (< 39 K) had higher odds of having children in all behavioural combination categories, apart from the High ST / High ED group (Table 3), than children in none or one risk behaviour (referent category) compared to parents of higher income.

**Table 2:** Univariate multinomial logistic regression analysis of factors associated with combinations of risk behaviours among children

	Low FV / high ED	High ST / Low FV	High ST / High ED	3 risk behaviours
Demographic				
Parental marital status (ref: Other)				
Married	0.33 (0.09, 1.24)	0.34 (0.08, 1.05)	0.31 (0.04, 2.06)	0.38 (0.10, 1.39)
Parent education (ref: GCSE or less)				
A-Level or post A-level equivalent	0.71 (0.10, 5.12)	0.36 (0.03, 5.11)	1.43 (0.10, 20.44)	0.83 (0.17, 4.06)
Degree level or above	0.81 (0.17, 3.81)	0.91 (0.16, 5.32)	0.61 (0.06, 6.58)	0.29 (0.08, 1.09)
Ethnicity (ref: Other)				
White / White British	1.24 (0.37, 4.09)	2.36 (0.46, 12.04)	2.18 (0.24, 20.04)	4.00 (0.82, 19.63)
Parental income (ref: more than 39 k in GBP per year)				
Less than 39 k per year	14.77 (1.62, 35.03)**	8.72 (0.82, 92.85)	32.00 (2.49, 51.43)**	20.36 (2.25. 84.59)**
Subjective SES (ref: low – less than median score of 7)	0.57 (0.20, 1.64)	0.78 (0.23, 2.59)	0.39 (0.08, 1.96)	0.62 (0.24, 1.89)
Behavioural				
Child eats breakfast while watching TV (ref: [2] or less days a week)	0.89 (0.24, 3.28)	3.50 (10.1, 12.22)*	10.02 (1.66, 60.20)**	2.00 (0.65, 6.13)
Child eats dinner while watching TV (ref: [2] or less days a week)	0.80 (0.14, 4.49)	1.23 (0.21, 7.12)	3.20 (0.49, 21.08)	3.29 (0.92, 11.85)
Child eats fruit and vegetables while watching TV (ref: [2] or less days a week)	0.79 (0.34, 4.05)	1.46 (0.37, 5.65)	10.03 (1.66, 60.20)**	2.40 (0.80, 7.23)
Child eats energy-dense snacks while watching TV (ref: [2] or less days a week)	4.78 (1.22, 18.70)*	2.56 (0.50, 13.07)	61.50 (5.85, 86.68)**	6.15 (1.65, 22.98)**
Parents’ TV/DVD viewing (ref: less than 2 h a day)	1.81 (0.64, 5.08)	17.50 (2.12, 44.67)**	3.12 (0.54, 17.84)	6.25 (1.83. 21.25)**
Parents’ energy-dense snack food consumption (ref: less than 1.4 a day)	12.80 (3.70, 44.30)***	2.00 (0.55, 7.32)	20.00 (2.07, 53.17)**	6.67 (2.21, 20.08)***
Physical environmental				
Home availability of energy-dense snack foods (ref: low – below median score of 8.5)	7.53 (2.31, 24.50)***	1.11 (0.32, 3.84)	13.29 (1.46, 26.99)*	5.90 (1.91, 18.29)**
Home availability of fruit and vegetables (ref: low – below a median score of 8)	0.32 (0.10, 1.05)	0.27 (0.08, 1.02)	1.11 (0.12, 10.64)	0.92 (0.24, 3.52)

Note: referent category for dependent variables is the none or one risk behaviour group

*p < 0.05; **p < 0.01; ***p < 0.001

**Table 3:** Multivariable multinomial logistic regression analysis of factors associated with combinations of risk behaviours among children

	Low FV / high ED	High ST / Low FV	High ST / High ED	3 risk behaviours
Demographic				
Parental income: Less than 39 k in GBP per year (ref: more than 39 k in GBP per year)	22.55 (1.39, 64.84)*	17.96 (1.04, 31.41)*	23.02 (0.70, 56.71)	31.38 (1.99, 93.62)*
Behavioural				
Child eats breakfast while watching TV (ref: [2] or less days a week)	0.78 (0.07, 9.01)	6.95 (1.10, 43.79)*	1.54 (0.06, 37.06)	3.05 (0.40, 23.06)
Child eats fruit and vegetables while watching TV (ref: [2] or less days a week)	0.27 (0.02, 4.63)	0.29 (0.03, 3.52)	0.65 (0.02, 21.82)	0.52 (0.05, 6.12)
Child eats energy-dense snacks while watching TV (ref: [2] or less days a week)	34.09 (2.02, 74.19)*	1.27 (0.08, 20.09)	10.55 (2.25, 45.02)*	8.79 (0.73, 106.79)
Parents’ TV/DVD viewing (ref: less than 2 h a day)	0.54 (0.09, 3.15)	27.01 (2.00, 71.26)*	1.47 (0.07, 30.81)	7.19 (1.07, 53.10)*
Parents’ energy-dense snack food consumption (ref: less than 1.4 a day)	25.18 (3.97, 59.51)***	1.33 (0.25, 7.16)	34.52 (1.94, 62.59)*	4.09 (0.74, 22.77)
Physical environmental				
Home availability of energy-dense snack foods (ref: low – below median score of 8.5)	7.66 (1.05, 30.61)*	1.46 (0.29, 7.23)	5.67 (0.34, 94.28)	5.82 (1.10, 30.67)*
Home availability of fruit and vegetables (ref: low – below a median score of 8)	0.32 (0.10, 1.05)	0.27 (0.08, 1.02)	1.11 (0.12, 10.64)	0.92 (0.24, 3.52)

Note: referent category of dependent variables is the none or one risk behaviour group

Only variables significant in the univariate analyses (Table 2) were entered into the multivariable analyses, hence not all variables are included in Table 3

*p < 0.05; **p < 0.01; ***p < 0.001

#### Behavioural factors

All but one of the behavioural factors (child eats dinner in front of the TV) were associated with an increased likelihood of risk factor behavioural combinations in the univariate analyses (Table 2).

In the multivariable model, children who ate breakfast at the TV on three or more days a week had higher odds of being in the High ST / Low FV group than in the none or one risk behaviour (referent category) compared to children who ate who ate breakfast at the TV on two or less days a week. Children who ate ED snacks at the TV on three or more days a week had higher odds of being in the Low FV / High ED and the High ST / High ED groups than children in none or one risk behaviour (referent category) compared to children who ate ED snacks at the TV on two or less days a week (Table 3).

Parents with high TV/DVD viewing had higher odds of having children in the High ST / Low FV and the three risk behaviour combination categories than children in none or one risk behaviour (referent category) compared to parents with lower TV/DVD viewing. Parents with high ED snack consumption had higher odds of having children in the Low FV / High ED and the High ST / High ED groups than children in none or one risk behaviour (referent category) compared to parents with lower ED snack consumption (Table 3).

#### Physical home environmental factors

Only home availability of ED snack foods was associated with behavioural risk factor combinations in the univariate analyses (Table 2). Parents who reported high availability of ED snack foods in the home had higher odds of having children in the Low FV / High ED and the three risk behaviour groups than children in none or one risk behaviour (referent category) compared to parents who reported lower availability of ED snack foods in the home (Table 3).

#### Health behaviour clusters

Table 4 describes the eight possible combinations of the three health behaviours examined and the observed and expected prevalence ratios of these health behaviours clusters. The majority of observed over expected ratios were close to 1 and ranged from 0.78 to 1.43. The three-risk behaviour combination of excessive ST, insufficient fruit and vegetable consumption, and high ED snack consumption occurred more frequently than expected (1.23 (0.89, 1.58)), as did the two-risk behaviour combination of high ED snack consumption and excessive ST, (1.43 (0.51, 2.35)), although non-significantly.

**Table 4:** Observed and expected prevalence of health risk behaviours, individually and in combination

No. of health behaviours	High TV/DVD	Low fruit and vegetable consumption	High energy-dense snack food consumption	O (%)	E (%)	O/E (95% CI)
3	x	x	x	14.77	12.03	1.23 (0.89, 1.58)
2	x	x	–	11.41	12.57	0.91 (0.60, 1.21)
	–	x	x	17.45	18.93	0.92 (0.69, 1.15)
	x	–	x	4.70	3.28	1.43 (0.51, 2.35)
1	x	–	–	2.68	3.43	0.78 (0.07, 1.49)
	–	x	–	24.83	19.79	1.25 (1.01, 1.49)
	–	–	x	6.71	5.16	1.30 (0.70, 1.90)
0	–	–	–	5.37	5.39	0.99 (0.41, 1.58)

*O* Observed prevalence, *E* Expected prevalence, *95% CI* 95% confidence interval, *X* Guideline not met, − Guideline met

## Discussion

The aim of this study was to examine the sociodemographic, behavioural, and home physical environmental correlates of co-occurring screen-time and unhealthy eating behaviours and to assess the clustering of screen-time and unhealthy dietary behaviours in young children. Given the attention that ST is increasingly receiving in the media and literature on children’s health, it is important to extend our knowledge about how other behaviours may co-occur and cluster with ST and what correlates seem to be important for such clusters. This allows a more nuanced approach to ST research.

While just under half of the children studied had none or only one risk behaviour, it is noteworthy that just under 20% had all three risk behaviours. This can be considered a large proportion and highlights a clear target for intervention. The cluster of low fruit and vegetable consumption, high ED snack consumption, and excessive ST and the two-risk cluster of high ED snack consumption and excessive ST both occurred more frequently than expected. Children engaging in too much ST also engaged in unhealthy eating behaviours. Although these findings did not attain significance, they are important for shaping the development of future interventions as they indicate the clustering, or co-occurrence, of unhealthy behaviours and suggest that behaviour change interventions might benefit from targeting ST and dietary behaviours concurrently or simultaneously. Given that evidence for the effectiveness of existing interventions which solely target sedentary behaviour is unconvincing [33], new interventions are required and our findings would suggest that these should target ST and diet together in efforts to bring about improved health behaviours. However, this would need to be guided by the data on the correlates of such behavioural clustering.

Our study showed that families of low household income, parents with high TV viewing, and parents reporting high availability of ED snacks in the home were at higher odds of having children in the three risk behaviour group than the none or one risk group. The finding that children from low income households were at higher odds of having all three risk behaviours is corroborated by those of Hardy et al. [17] who found evidence of clustering of sedentary behaviour, physical activity and dietary behaviours in Australian adolescents, particularly among adolescents from low income households. Our findings show that such relationships start early in childhood in this UK based sample. It has been known for some time that socio-economic status (SES) is associated with higher levels of TV viewing [34] and also with poorer dietary intake [35]. However, how to address this issue has been shown to be more problematic. Low SES might be associated with more barriers to be outside of the home, such as greater crime in the local area or less open and green space, hence increasing the likelihood of greater indoor sedentary time. Cost is also a recognised barrier to parents purchasing healthy foods for their children [35, 36]. While these barriers are acknowledged, few interventions have specifically addressed the issue of SES in relation to poor diet and ST in the home, yet the current findings add further weight to this group being in need of support.

The finding that parents with higher TV viewing were at higher odds of were at higher odds of having children in the three risk behaviour group than the none or one risk group aligns with the findings of Cameron et al. [19], who showed that clusters of unhealthy behaviours were concordant in mothers and their children, particularly those defined by sedentary behaviours and consumption of ED food and drink. The associations between parental modelling of individual healthy (e.g. physical activity [37] and consumption of fruits and vegetables [15]) and unhealthy (e.g. high ST [14] and ED snack food consumption [38]) behaviours and child behaviours are well established in the literature, and our findings that parental modelling of TV viewing is associated with the co-occurrence of 3 unhealthy behaviors is novel and further develops this literature. Our findings suggest that parental TV viewing behaviour has the potential to transfer across to different health behaviours in children. Strategies to un-couple unhealthy behaviours need to address parental as well as child behaviours and should focus on the creation of a healthy eating and active home environment, screen-free eating occasions, parents modelling healthy behaviours (i.e. switching off screens when with children) and finding opportunities for whole family active alternates to sedentary screen-based activities.

Higher availability of ED snacks in the home increased the odds of having children who exhibited the co-occurrence of all three risk behaviours. This may be a surrogate measure for the availability of less healthy foods within the household. Moreover, children’s consumption of ED snacks in front of the TV was also associated with the Low FV/High ED and High ST/High ED pattern, suggesting that ED snacking is problematic in 5–6-year-olds and another important target for intervention. Not only might different food shopping strategies be needed, but healthy and palatable alternatives are also required (e.g., chopped ready prepared fruit). Parents, particularly those from low SES backgrounds, might benefit from advice regarding cost effective ways to purchase healthy foods (e.g., buying frozen or tinned foods as a way to reduce waste), as cost and waste are important predictors of parents not repeatedly offering healthy foods to their children [36].

It is also interesting that we found that children eating breakfast in front of the TV on three or more days a week were at higher odds of being in the High ST/Low FV group than the non or one risk behaviour group compared with those who ate breakfast at the TV on two or less days a week. Previous research has demonstrated an underlying association between TV viewing and lower intakes of fruit and vegetable consumption [39]. Eating meals such as breakfast in front of the TV has been linked to lower intakes of fruits and vegetables and higher intakes of unhealthy foods [40]. Distractions, like the TV, have been shown to increase food intake [41] and TV viewing might be a strategy employed by parents in an effort to facilitate breakfast intake during busy morning routines. Eating breakfast in front of the TV could reduce opportunities for eating as a family, which is important for an array of health behaviours [42]. Furthermore, evidence suggests that SES is inversely related to eating in front of the TV (e.g. [43]) and so this reflects another difference in children’s health behaviours among SES groups. Given the young age of the children in our study, eating breakfast away from screens represents another area to be targeted in future.

The findings of this research are valuable for helping to inform the development of future health-promotion interventions. This research has highlighted clusters and patterns of high-risk health behaviours in young children which warrant targeting in future programmes aiming to reduce childhood overweight/obesity and improve children’s activity levels and healthy eating behaviours. Strengths of this research include the assessment of multiple risk factors and analysis of co-occurrence of these behaviours and the use of a sample of parents of young children. Limitations include the fairly homogenous sample and low response rate, which limits generalisability, the moderate sample size, and the cross-sectional nature of the data. The accuracy of the parent-reported child food frequency may be impacted especially for parents whose children eat school lunches and may therefore not be aware/sure of what/how much their children are eating and could potentially have led to an under or over-reporting of food items. Furthermore, the adaptations made to the ASAQ to assess children’s screen-based behaviours may have impacted on the reliability and validity of the measure and could have led to inflated/deflated values for reported ST. Caution is required in interpreting our findings in light of these limitations.

## Conclusion

In conclusion, parents’ own behaviour has been identified as an important influence on the co-occurrence of ST and unhealthy dietary behaviours in children as young as 5 years. Uniquely, our findings extend past research to highlight the co-occurrence and clustering of multiple health risk behaviours in young children. There is therefore a need for further research into the development of behavioural clustering in young children to identify and further understand the mechanisms underlying the synergy among health behaviours. Feasibility interventions that promote reductions in both ST and unhealthy dietary behaviours reciprocally, while simultaneously focusing on changing parental behaviours, are warranted.

## Additional file

Additional file 1: **Table S1.** Description and distribution (%) of demographic, behavioural, home physical environmental variables (DOC 60 kb)

## Abbreviations

ED snacks: Energy-dense snacks
FV: Fruit and vegetables
SB: Sedentary behaviour
ST: Screen-time
TV: Television viewing
