# Supplementary material for: Clustering and correlates of screen-time and eating behaviours among young children
Source: BMC Public Health. 2018 Jun 18;18:753. doi: 10.1186/s12889-018-5698-9 (PMC6006584; doi:10.1186/s12889-018-5698-9)
Supplement: Supplementary file 1 — Table S1. Description and distribution (%) of demographic, behavioural, home physical environmental variables (DOC 60 kb) [file 12889_2018_5698_MOESM1_ESM.doc]

Table S1. Description and distribution (%) of demographic, behavioural, home physical environmental variables

|  | **Total (n=126)** |
| --- | --- |
| **Demographic** | |
| ***Child gender*** |  |
| Male | 49 |
| Female | 51 |
| ***Child age*** (mean (SD)) | 5.58 (0.73) |
| ***Parent gender*** |  |
| Female (mother) | 81.9 |
| Male (father) | 16.1 |
| ***Parent age*** (range from 25-52 years, mean (SD)) | 38.61 (4.97) |
| ***Parental marital status*** |  |
| Married | 80.5 |
| Other | 19.5 |
| ***Parent education*** |  |
| GCSE or less | 12.8 |
| A-Level or post A-level equivalent | 18.2 |
| Degree level or above | 68.3 |
| ***Ethnicity*** |  |
| White / White British | 80.5 |
| Other | 19.5 |
| ***Parental income*** |  |
| 39k or less per year | 22.4 |
| More than 39k a year | 77.6 |
| ***Subjective SES*** (scale from 1-10, median: 7) |  |
| Low | 40.9 |
| High | 59.1 |
| **Child behavioural** | |
| Child eats breakfast while watching TV^1^ |  |
| Low | 70.8 |
| High | 29.2 |
| Child eats dinner while watching TV^1^ |  |
| Low | 83.3 |
| High | 16.7 |
| Child eats fruit and vegetables while watching TV^1^ |  |
| Low | 70.8 |
| High | 29.2 |
| Child eats energy-dense snacks while watching TV^1^ |  |
| Low | 75 |
| High | 25 |
| Parents TV/DVD viewing (categorised at 2 hours per day) |  |
| Low | 36.5 |
| High | 63.5 |
| Parents energy-dense snack food consumption (categorised at median of 1.4 a day) |  |
| Low | 56.5 |
| High | 43.5 |
| **Physical environment** | |
| Home availability of energy-dense snack foods (median score: 8.5) |  |
| Low | 50 |
| High | 50 |
| Home availability of fruit and vegetables (median score: 8.0) |  |
| Low | 22.1 |
| High | 77.9 |

^1^Low = ‘2 or less days a week’; High = ‘3 or more days a week’

^2^Low = less than twice a week’; High = ‘2 or more times a week’
